# The Effect of Meditation-Based Interventions on Patients with Fatigue Symptoms: A Systematic Review and Meta-Analysis

**DOI:** 10.5334/pb.1182

**Published:** 2023-06-23

**Authors:** Florence Requier, Harriet Demnitz-King, Tim Whitfield, Olga Klimecki, Natalie L. Marchant, Fabienne Collette

**Affiliations:** 1 GIGA-CRC In Vivo Imaging, Université de Liège, Bâtiment B30, Allée du Six Août, 8, Sart Tilman, 4000 Liège, Belgium; 2 Department of Psychology, Cognition and Behavior, University of Liège, Liège, Belgium; 3 Division of Psychiatry, University College London, London, UK; 4 Swiss Center for Affective Sciences, University of Geneva, Geneva, Switzerland; 5 Clinical Psychology and Behavioral Neuroscience, Faculty of Psychology, Technische Universität Dresden, Dresden, Germany

**Keywords:** meditation, mindfulness, compassion, non-pharmacological intervention, systematic review, meta-analysis, fatigue

## Abstract

Persistent fatigue constitutes a prevalent and debilitating symptom in several diseases. The symptom is not effectively alleviated by pharmaceutical treatments, and meditation has been proposed as a non-pharmacological intervention. Indeed, meditation has been shown to reduce inflammatory/immune problems, pain, stress, anxiety and depression which are associated with pathological fatigue. This review synthesizes data from randomized control trials (RCTs) that explored the effect of meditation-based interventions (MeBIs) on fatigue in pathological conditions. Eight databases were searched from inception to April 2020. Thirty-four RCTs met eligibility criteria and covered six conditions (68% cancer), 32 of which were included in the meta-analysis. The main analysis showed an effect in favor of MeBIs compared to control groups (*g* = 0.62). Separate moderator analyses assessing control group, pathological condition, and MeBI type, highlighted a significantly moderating role of the control group. Indeed, compared to actively controlled studies, studies using a passive control group were associated with a statistically significantly more beneficial impact of the MeBIs (*g* = 0.83). These results indicate that MeBIs alleviate pathological fatigue and it seems that the studies with a passive control group showed a greater effect of MeBI on the reduction of fatigue compared to studies using active control groups. However, the specific effect of meditation type and pathological condition should be analyzed with more studies, and there remains a need to assess meditation effects on different types of fatigue (i.e., physical and mental) and in additional conditions (e.g., post-COVID-19).

## Introduction

In the field of neurological and psychiatric disorders, fatigue is typically defined as extreme and persistent mental and/or physical tiredness, weakness or exhaustion (Dittner, Wessely, & Brown, 2004). This subjective symptom refers both to physical fatigue that concerns neuromuscular and muscle failure, and mental fatigue that relates to cognitive exhaustion after a mental effort, as well as the efficiency of mental workload in the absence of cognitive failure or motor weakness (Chaudhuri & Behan, 2000, 2004; Wylie & Flashman, 2017). Fatigue has repercussions on work but also on daily life activities (Bakshi, 2003; Duncan, Wu, & Mead, 2012; Morrow et al., 2002; Ponsford et al., 2014; Rosenthal et al., 2008).

Fatigue, both mental and physical, is observed transiently in the healthy population, typically due to prolonged periods of demanding activities requiring sustained efficiency, whereas it manifests in a more continuous and severe way in a large number of illnesses (Penner & Paul, 2017). Pathological fatigue is characterized by its severity, its persistence and excessively disabling impact on patients’ daily life (Christodoulou, 2005). According to Penner and Paul, the factors that contribute to fatigue are highly interdependent, and consequently fatigue is a complex condition that can emerge as a symptom or a comorbidity of neurological or systemic diseases. The authors proposed to distinguish between primary and secondary fatigue. Primary fatigue refers to fatigue symptoms emerging from the disease itself (e.g., from cytokine release and inflammatory processes). Secondary fatigue results from concomitant factors associated to the disease, such as sleep disorders, depression, pain and pharmacological treatments. However, fatigue can also be considered as a comorbid factor (e.g., in association with anxiety, depression or sleep disorders). The prevalence of the fatigue symptom is greater than 60% in some pathological conditions, including cancer (Joly et al., 2019), multiple sclerosis (Kluger, Krupp, & Enoka, 2013; for a review, see Immink, 2014), stroke (Immink, 2014; Lerdal et al., 2009), traumatic brain injury (Bushnik, Englander, & Wright, 2008; for a review, see Immink, 2014), and kidney disease (Artom et al., 2014). Moreover, high levels of fatigue primarily characterizes conditions such as myalgic encephalomyelitis (also known as chronic fatigue syndrome) and fibromyalgia (Schweitzer et al., 1995; Vincent et al., 2013). Very recent publications also report fatigue complaints in long-COVID. A meta-analysis showed 32% of 25,268 patients still have persistent fatigue more than 12 months after the infection (Ceban et al., 2022).

Currently there is an absence of convincing evidence for a pharmacological treatment for fatigue (Braley & Chervin, 2010; Penner & Paul, 2017). Intervention programs based on meditation practice have recently received growing attention among health professionals. Meditation is a broad term for a series of practices that can overlap (Dahl, Lutz, & Davidson, 2015; Hofmann, Grossman, & Hinton, 2011). Among them, mindfulness-based interventions develop mainly meta-awareness and self-regulation processes, while loving-kindness-based and compassion-based interventions encourage the cultivation of positive attitudes towards oneself and others.^1^ Consequently, it is considered that meditation training teaches individuals to develop attentional and emotional self-regulation, resulting in less reactivity to negative experiences (Tang, Hölzel, & Posner, 2015). In healthy individuals, meditation-based interventions (MeBIs) generally improve quality of life (Khoury et al., 2015), with lower levels of stress, anxiety and depression (Manning & Steffens, 2018). In disease states, MeBIs are associated with reduced depression and anxiety symptoms (O’Connor, Piet, & Hougaard, 2014) and better pain management (Morone et al., 2008). Consequently, MeBI could be considered as an alternative, non-pharmacological approach to relieve fatigue symptoms associated with different pathological conditions.

We identified two systematic reviews assessing the impact of mindfulness-based interventions on fatigue in neurological disorders (Immink, 2014; Ulrichsen et al., 2016). A limited evidence was observed in Immink (2014), while Ulrichsen et al. (2016) reported a moderately sized (*g* = –0.37) evidence in favor of mindfulness-based interventions for fatigue reduction. Further, two meta-analyses have evaluated the effect of mindfulness-based interventions on fatigue levels in breast cancer patients (Castanhel & Liberali, 2018; Haller et al., 2017), and another was interested in the effect of MeBIs on psychological well-being in cancer survivors included fatigue outcomes (Johns et al., 2021). These three reviews reported moderate, small and large effect sizes, respectively. However, there are limitations to these syntheses. No meta-analysis made a clear distinction between the different kinds of MeBI, nor between cognitive and physical fatigue. So far, there is limited evidence on the effects of MeBIs on mental fatigue. For instance, only one of 57 studies identified by a review investigating the effects of therapeutic interventions on mental fatigue following traumatic brain injury (Wylie & Flashman, 2017) used a MeBI approach (Johansson, Bjuhr, & Rönnbäck, 2012). This study showed that the group assigned to mindfulness-based stress reduction experienced a reduction in self-reported mental fatigue in comparison to a waitlist control group (who received no active treatment).

Furthermore, to determine the relative efficacy of MeBIs for reducing fatigue versus other non-pharmacological interventions is of interest. Physical activity programs have a moderate positive effect in multiple sclerosis according to the meta-analysis of Heine et al. (2015) but no effect was found in a study on patients with traumatic brain injury (Bateman et al., 2001). Melatonin (Grima et al., 2018) or light therapy (Sinclair et al., 2014) showed a positive effect with alleviation of fatigue symptoms in patients with traumatic brain injury, but the effect does not persist after the end of the light therapy intervention (Sinclair et al., 2014) while the data are not available for melatonin treatment. However, up to now, the most relevant intervention seems to be cognitive-behavioral therapy (CBT) (for a study on multiple sclerosis: Van Kessel et al., 2008; for studies on traumatic brain injury: Nguyen et al., 2017; Nguyen et al., 2019), with a maintenance of effect up to 4 months after the end of the intervention in post-stroke patients (Nguyen et al., 2017; Nguyen et al., 2019). It is proposed that CBT act directly on fatigue symptoms or can act on emotional symptoms (depression, anxiety) linked with the fatigue state (Van Kessel et al., 2008; Nguyen et al., 2017). Moreover, a meta-analysis of randomized controlled trials (RCTs) evaluating CBT for myalgic encephalomyelitis identified a large effect of CBTs on physical fatigue and a small effect of CBTs on mental fatigue (Malouff et al., 2008). Therefore, meditation, which is also known to reduce negative emotional feelings, may also have a positive effect on fatigue.

The primary aim of this work is to provide an up-to-date systematic review and meta-analysis of the effect of MeBIs on fatigue symptoms in pathological conditions. To this end, we investigated the effect of MeBIs on fatigue in neurological disorders (e.g., multiple sclerosis, stroke, traumatic brain injury), and other pathological conditions in which fatigue is one of the main symptoms, such as cancer, myalgic encephalomyelitis, fibromyalgia, or kidney disease. First, we determined if the effect of a MeBI is larger than the effect of a control group. Then, when data were available, we looked if MeBI differently impacted fatigue regarding the type of MeBI used, the type of fatigue assessed (physical, mental), the pathological condition studied, or the comparator chosen (passive vs active control group).

## Methods

### Registration

In line with the Preferred Reporting Items for Systematic Review and Meta-Analysis Protocols (PRISMA-P) guidelines (Page et al., 2021), the protocol for the present review was registered on Prospero (CRD42020187836).

### Eligibility criteria

Concerning the inclusion criteria, we searched for published and unpublished studies that randomized participants to either a meditation-based intervention or control condition(s). Samples had to comprise adults (mean age ≥ 18 years) with a pathological condition in which fatigue is considered as a debilitating symptom (e.g., multiple sclerosis, stroke, traumatic brain injury, cancer, fibromyalgia, myalgic encephalomyelitis) (Artom et al., 2014; Hjollund, Andersen, & Bech, 2007; Johns et al., 2021; Penner & Paul, 2017). The primarily studied intervention must have been described clearly enough to be classified as a mindfulness- or compassion-based intervention, and not an alternative form of practice (such as mantra meditation) or intervention (e.g., Cognitive Behavioral Therapy, Yoga). The MeBI must have included mindfulness and/or compassion practices as the central component, and been delivered (i) over four or more sessions (benefits of a brief meditation training were already observed after three and four sessions regarding psychological (Creswell et al., 2014) and cognitive (Zeidan et al., 2010) aspects, respectively); (ii) in person or remotely; (iii) to groups or individuals. The meditation condition had to be compared to at least one active control condition(s) (e.g., CBT or health educational intervention) and/or passive control condition (including no intervention and waitlist). To be eligible, outcomes had to be self-reported measures of subjective fatigue and be administered before and after the intervention. Moreover, outcomes had to be standardized scales (or subscales). If a study did not provide the necessary data, then the authors were contacted.

Concerning the exclusion criteria, we decided not to include studies (i) where the MeBI was delivered in a retreat setting or solely comprised mantras; (ii) only reporting on brain activity or other biological outcomes; (iii) focusing on psychological disorders (such as burnout, post-traumatic stress disorder, psychosis) or medical conditions where fatigue is not considered as a main complaint associated with the disease (obesity, cardiac disorders).

### Search strategy

Searches were conducted on eight databases (Ovid: Medline, PsycInfo, EMBASE, AMED, as well as Scopus, CINAHL, Google Scholar, and Web of Sciences) to identify eligible studies. We also surveyed gray literature (OpenGrey, ProQuest). Searches were conducted in April 2020, and articles were independently screened by two reviewers at the title-abstract and full-text stages. Further, the reference lists of articles and reviews were hand searched for eligible studies. Search terms included ‘Meditation’, ‘Mindfulness’, ‘Mindfulness-Based Interventions’, ‘Fatigue’, ‘Fatigue Syndrome’, ‘Chronic’ (more details in Table 1).

**Table 1:** Search terms in Medline on the Ovid platform.


MEDLINE (OVID PLATFORM)

**1.** Mindfulness/

**2.** Meditation/

**3.** (medit* or mindful*).ti,ab,kf

**4.** 1 or 2 or 3

**5.** exp Fatigue/or Fatigue Syndrome, Chronic/

**6.** (fatig* or exhaust* or tired*).ti,ab,kf

**7.** 5 or 6

**8.** 4 and 7


### Data extraction

Three reviewers (HDK, TW, FR) independently extracted data in pairs from eligible papers using the same template and with a specific coding. The template included: (i) demographic details, (ii) pathological condition, (iii) fatigue type, (iv) intervention related details, and (v) effect sizes. Any disagreements were resolved by consensus.

### Coding scheme

There were two categories regarding the types of comparators: passive and active control groups. If there was more than one control group, the active control was selected over the passive control group. When two active groups were included, we selected the one most comparable to the MeBI. Four MeBI types were highlighted: (1) Mindfulness interventions *characterized by attentional/emotional regulation and insight practices* (representing studies which used a general mindfulness MeBI, as well as a mindfulness-based stress reduction intervention or a mindfulness-based cognitive therapy), (2) Compassion (including loving-kindness) MeBI (*related to the ability to develop positive attitudes towards people*) administered alone or with the addition of Mindfulness components (which concerned the studies which used Compassion during one session or more, as Compassion MeBI highly depends on mindfulness components (Hofmann et al., 2011), (3) Remote MeBI (studies in which meditation was delivered via an application, via the Internet or by phone) and (4) Other (which included Tibetan Sound Meditation Program, Brain Wave Vibration meditation, Mindfulness-Based Art Therapy).

Concerning the types of pathological conditions, there were six categories: cancer, multiple sclerosis, myalgic encephalomyelitis, fibromyalgia, acute brain injury (i.e., stroke and traumatic brain injury), and kidney disease.

### Risk of bias

Two reviewers independently assessed the risk of bias and integrity of the randomization processes using the Cochrane risk of bias tool version 2 (Sterne et al., 2019) and the quality of evidence using the Grading of Recommendations, Assessment, Development, and Evaluation (GRADE) Methodology (training.cochrane.org/resource/grade-handbook).

### Meta-analysis

Our strategy was to calculate the meta-analysis scores by comparing effect sizes within and between studies. We used Hedges’ Standardized Mean Difference and ran a random-effect meta-analysis using the default restricted maximum likelihood as estimator (Borenstein et al., 2009). For outcomes where lower scores indicate less fatigue, we multiplied values by –1; thus, lower scores always indicate more fatigue. For interpretation of effect sizes, we utilized Lipsey & Wilson’s (1993) categories: *g* ≤ 0.32 corresponds to a small effect size, 0.33 ≤ *g* ≤ 0.55 to a medium effect size, and *g* ≥ 0.56 to a large effect size.

For the meta-regression, we report models using the Hartung-Knapp method (van Aert & Jackson, 2019). The first model did not include any moderators, and thus simply reports the pooled effect size. For the other models, we selected three moderators: type of control group, type of MeBI, and type of pathological condition.

### Exploratory subgroup analyses

For the subgroup analyses, we looked in each modality of the different moderators if there was a significant difference between meditation-based interventions and control groups. In the first subgroup analysis, we separately evaluated actively controlled and passively controlled studies. We next calculated pooled effect sizes for each type of MeBI. Finally, we analyzed the effect of MeBI for each type of pathological condition separately.

### Heterogeneity analyses

For heterogeneity concerning meta-analysis and subgroups analyses, we evaluated two parameters: tau^2^, which represents the variance between the studies, and I^2^ (>75% indicates substantial heterogeneity), which refers to the proportion of observed dispersion due to real variation in effect sizes, rather than random error. R version 3.4.4 (R foundation for statistical computing) was used to perform all analyses.

## Results

### Study selection

**Figure 1:**
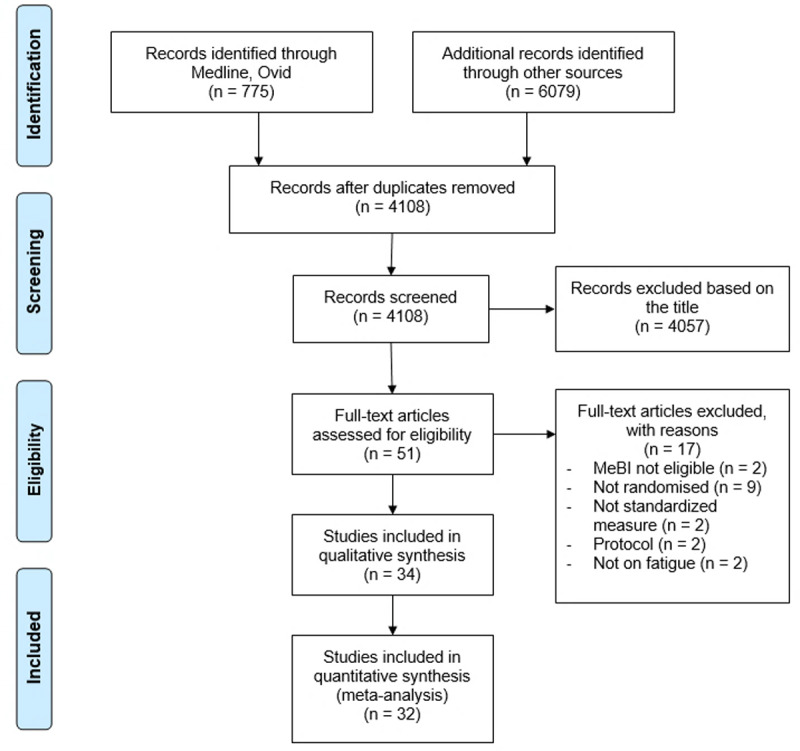
PRISMA flowchart with the different studies (MeBI: Meditation-Based Intervention). *Note*: Adapted from Moher et al. (2009).

The literature search (inception to April 2020) yielded 6854 records; after the removal of duplicates (*k* = 2746), 4108 studies remained. Following title and abstract screening, 4057 studies were excluded. Through the full-text screening of the 51 remaining records, we excluded 17 studies. The kappa index for the whole screening was .94. This resulted in a final total of 34 studies meeting the eligibility criteria (Figure 1).

### Characteristics of included studies

The results are ordered by kinds of pathological condition and by kinds of control groups in Table 2.

**Table 2:** Results table ordered by pathologies and by kinds of control groups (active then passive).


PATHOLOGICAL CONDITIONS	STUDY, DATE	INTERVENTION AS STATED IN THE STUDIES	CONTROL GROUP	ASSESSMENT

**Cancer**	**Bruggeman-Everts et al., 2017**	*Web-based MBCT* (N = 55)^3^	*AAF (N = 62)*	Checklist Individual Strength (CIS-F)

	**Carlson et al., 2016**	*Mindfulness-Based Cancer Recovery* (N = 134)^1^	*SET (N = 118)*	The Profile of Mood States (POMS) – fatigue

	**Johns et al., 2016**	*MBSR* (N = 35)^2^	*PES (N = 36)*	Fatigue Symptom Inventory (FSI)

	**Gok Metin et al., 2019**	*Mindfulness Meditation* (N = 33)^1^	*PMR (N = 30)*	Brief Fatigue Inventory (BFI)

	**Witek Janusek et al., 2019**	*MBSR* (N = 84)^1^	*Health Education (N = 80)*	Multidimensional Fatigue Scale Inventory – Short Form (MFSI-SF)

	**Wren et al., 2019**	*Lovingkindness Meditation* (N = 23)^2^	*Music (N = 16)*	FACIT Fatigue scale

	**Blaes et al., 2016**	*Mindfulness-Based Cancer Recovery* (N = 28)^1^	*Waitlist/TAU (N = 13)*	Functional Assessment in Cancer Therapy-Fatigue (FACT-F)

	**Bower et al., 2015**	*Mindful Awareness Practices program* (N = 39)^1^	*Waitlist (N = 32)*	Fatigue Symptom Inventory (FSI) – total

	**Dodds et al., 2015**	*Cognitively-Based Compassion Training* (N = 12)^2^	*Waitlist (N = 16)*	Medical Outcomes Study Short Form 12-Item Health Survey (SF-12)

	**Hoffman et al., 2012**	*MBSR* (N = 103)^1^	*Waitlist (N = 111)*	The Profile of Mood States (POMS) – fatigue

	**Jang, 2016**	*Mindfulness-based art therapy* (N = 12)^4^	*Waitlist (N = 12)*	European Organization for Research and Treatment of Cancer Quality of Life Questionnaire

	**Johns et al., 2015**	*MBSR for cancer related fatigue* (N = 18)^2^	*Waitlist (N = 17)*	Fatigue Symptom Inventory (FSI)

	**Kim et al., 2013**	*Brain Wave Vibration meditation* (N = 51)^4^	*TAU (N = 51)*	Revised Piper Fatigue scale (PFS)

	**Kubo et al., 2019**	*Headspace smartphone app* (N = 40)^3^	*Waitlist (N = 32)*	Brief Fatigue Inventory (BFI)

	**Lengacher et al., 2012**	*MBSR* (N = 41)^1^	*TAU (N = 43)*	M.D. Anderson Symptom Inventory (MDASI)

	**Lengacher et al., 2016**	*MBSR for Breast Cancer* (N = 167)^1^	*Waitlist (N = 155)*	Fatigue Symptom Inventory (FSI) – severity

	**Liu et al., 2019**	*MBSR* (N = 49)^1^	*TAU (N = 53)*	QoL Questionnaire Core 30Items (QLQ-C30)

	**Meiklejon, 2008**	*Shortened MBSR* (N = 26)^1^	*Waitlist (N = 21)*	The Profile of Mood States (POMS) – fatigue

	**Messer et al., 2019**	*Internet-delivered mindfulness treatment* (N = 11)^3^	*TAU (N = 10)*	The Fatigue Symptom Inventory (FSI) – total

	**Milbury et al., 2013**	*Tibetan Sound Meditation program* (N = 23)^4^	*Waitlist (N = 24)*	Brief Fatigue Inventory (BFI)

	**Park et al., 2020**	*MBCT* (N = 38)^1^	*Waitlist (N = 36)*	Brief Fatigue Inventory (BFI)

	**Speca et al., 2000**	*Mindfulness Meditation-Based Stress Reduction Program* (N = 53)^1^	*Waitlist (N = 37)*	The Profile of Mood States (POMS) – fatigue

	**Van Der Lee et al., 2012**	*MBCT* (N = 59)^1^	*Waitlist (N = 24)*	Checklist Individual Strength (CIS)

**Multiple sclerosis**	**Carletto et al., 2017**	*Body-Affective Mindfulness Intervention* (N = 45)^2^	*Psycho-Educational Intervention (N = 45)*	Fatigue Severity Scale (FSS)

	**Cavalera et al., 2019**	*Online MBSR* (N = 54)^3^	*Online psychoeducation treatment (N = 67)*	Modified Fatigue Impact Scale (MFIS)

	**Senders et al., 2019**	*MBSR* (N = 33)^2^	*MS Education (N = 29)*	PROMIS

	Grossman et al., 2010	*Mindfulness-Based Intervention* (N = 76)^1^	*TAU (N = 74)*	Modified Fatigue Impact Scale (MFIS)

	**Bogosian et al., 2015**	*Adapted Skype Distant-Delivered Mindfulness Intervention* (N = 19)^3^	*Waitlist (N = 21)*	Fatigue Severity Scale (FSS)

	**Simpson et al., 2017**	*MBSR* (N = 25)^1^	*Waitlist (N = 25)*	Modified Fatigue Impact Scale (MFIS)

**Myalgic encephalomyelitis**	**Rimes et al., 2013**	*MBCT* (N = 15)^1^	*Waitlist (N = 19)*	Chalder Fatigue Scale

	**Surawy et al., 2005**	*Mindfulness-based program based on MBSR and MBCT* (N = 9)^1^	*TAU (N = 8)*	Chalder fatigue scale

**Fibromyalgia**	**Cash et al., 2015**	*MBSR* (N = 51)^1^	*Waitlist (N = 40)*	Fatigue Symptom Inventory (FSI) – total

**Acute brain injury**	**Johansson et al., 2012**	*MBSR* (N = 12)^1^	*Waitlist (N = 14)*	Mental fatigue Scale (MFS)

**Kidney disease**	**Gross et al., 2017**	*Telephone-adapted MBSR* (N = 27)^3^	*Telephone-Based Support Group (N = 28)*	Patient-Reported Outcomes Measurement Information System (PROMIS)


*Note*: MBCT: Mindfulness-Based Cognitive Therapy; AAF: Ambulant Activity Feedback therapy; SET: Supportive Expressive Therapy; MBSR: Mindfulness Based Stress Reduction; PES: PsychoEducation and Support; PMR: Progressive Muscle Relaxation procedure; TAU: Treatment As Usual; MS: Multiple Sclerosis; ^1-4^ labelling of the interventions according to the literature: ^1^ Mindfulness-based interventions, ^2^ Compassion MeBI only or with Mindfulness components, ^3^ Delivered remotely, ^4^ Other type of meditation.

Our selection process resulted in a total of 34 studies. Publication year ranged from 2000 to 2020 (median: 2016). The majority of studies took place in the United States (*k =* 15; 44%). Six studies took place in the United Kingdom (18%); two studies in Italy (6%), Korea (6%), Netherlands (6%), and Canada (6%); and one study in China (3%), Turkey (3%), Japan (3%), Switzerland (3%), and Sweden (3%).

The median number of participants by study was 36.5 for the meditation arm and 31.5 for the control arm. In 32 studies, age is reported, and the participants were mainly middle-aged (mean age = 51.80, standard deviation = 4.73; range: 42–58). There was a high proportion of women (84.61%), which can partially be explained by some studies focusing on breast cancer in women.

Among various pathologies, cancer (*k* = 23) was the main topic, followed by multiple sclerosis (*k* = 6). Other pathologies included myalgic encephalomyelitis (*k* = 2), fibromyalgia (*k* = 1), acute brain injury (*k* = 1), and kidney disease (*k* = 1).

Among the 34 selected studies, 16 different scales were used. The Fatigue Symptom Inventory was the most common scale used (e.g., studies on fibromyalgia or cancer; see Table 2 and *Supplemental Table 1*). As detailed in Supplemental Table 1, some questionnaires assessed a general fatigue state with one or several questions, while others distinguished between mental and physical fatigue (however, only global scores were reported). Only one study used a mental fatigue scale (Johansson et al., 2012).

The included studies (Table 2) featured various types of MeBI, which were categorized in four groups (see *Supplemental Table 2*). Most of the studies used mindfulness-based interventions. More specifically, 18 studies used Mindfulness-based reduction interventions or derivative forms of this intervention. Seven studies used Compassion interventions or Mindfulness and Compassion interventions; more specifically, one study used a compassion intervention only (Wren et al., 2019). Six studies gave a mindfulness MeBI delivered remotely. Three studies comprised other types of MeBI: Tibetan Sound Meditation Program, Brain Wave Vibration meditation, or Mindfulness-Based Art Therapy. Interventions lasted around eight weeks (*k* = 33, minimum: 6 weeks, maximum: 12 weeks) and had a median of eight meditation sessions (*k* = 31, minimum: 6 sessions, maximum: 84 sessions). The duration of each session had a median of 120 minutes (*k* = 27, minimum: 20 minutes, maximum: 180 minutes). Adherence data have not been reported due to the limited amount of data available and the great variability in the way they were reported between studies. For example, six studies reported percentages of people practicing at home without providing duration of practice and ten provided an average of the number of minutes spent by people to do some exercises at home.

**Figure 2:**
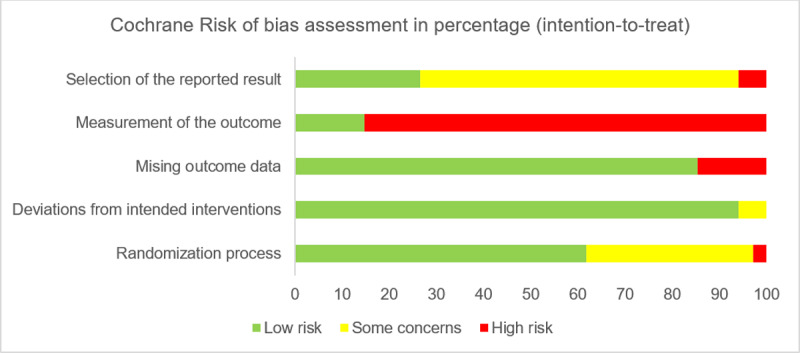
Cochrane Risk of bias assessment. The graph presents ratings for all 34 studies included in the systematic review.

Among the 34 studies, ten (29%) had an active control group and 24 (71%) had a passive control group. Four of the ten studies with an active control group had more than one control group. Three studies (9%) had an active control group and a passive control group, and one study (3%) had two active control groups. The control interventions in the nine actively controlled studies used in the meta-analysis were: Ambulant Activity Feedback therapy, Health Education, Music, Multiple Sclerosis Education, Progressive Muscle Relaxation procedure, Psycho-Educational Intervention, Telephone-Based Support Group, Online psychoeducation treatment, Psychoeducation and support. For the 23 passively controlled studies used in the meta-analysis, 17 (74%) used a waitlist and six (26%) utilized usual care.

### Immediate and follow-up effects of the interventions

Of the 34 studies included, 26 (76%) reported a significant decrease of fatigue symptoms immediately after the intervention. Of these 26 studies, three showed that the effect is superior to the effect of another active intervention and 18 showed a superiority effect by comparison to passive control. The five remaining studies showed no differences between MeBI and a control group (one passive and four active). Sixteen studies with a positive immediate effect of MeBI reported long-term follow-up (from 10 to 64 weeks after the end of the intervention). From these studies, the positive effect of MeBI was maintained at long-term for nine studies (56%). More precisely, the maintenance of the effect was superior to the one in the passive control groups in seven studies out of the nine and for two, MeBI did not show a better maintenance than control group at follow-up (see *Supplemental Table 3 for a detailed presentation*).

**Figure 3:**
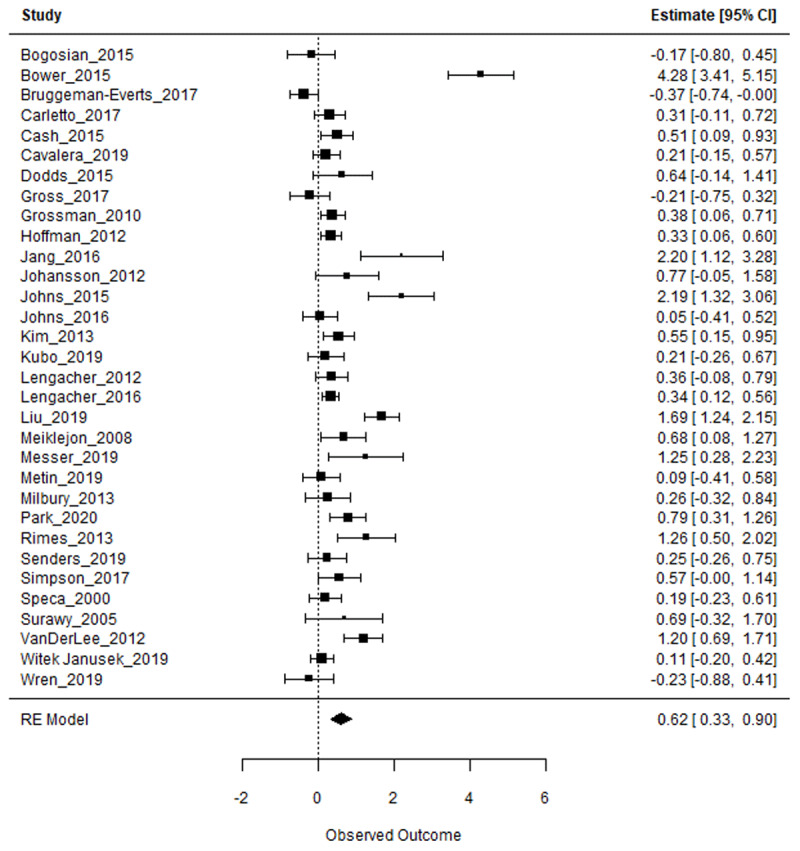
Forest plot of the standardized mean differences of all studies (negative values correspond to evidence in favor of the control group and positive values represent evidence in favor of the MeBI group, CI: Confidence Interval, RE: random-effects).

### Risk of bias

The risk of bias assessment of 34 studies is summarized according to the five domains of the assessment tool (Figure 2).

Concerning the first domain of ‘Randomization process’, the majority of studies were judged to be at low risk of bias (61.8%; *k* = 21), some were judged to have some concerns (35.3%; *k* = 12), and the remainder were considered to be at high risk of bias (2.9%; *k* = 1).

For the second domain, ‘Deviations from intended interventions’, the majority of studies were judged to be at low risk of bias (94.1%; *k* = 32). The remaining studies were considered to have some concerns (5.9%; *k* = 2).

Related to the third domain (‘Missing outcome data’), the majority of studies were judged to be at low risk of bias (85.3%; *k* = 29), with the remaining studies considered to be at high risk (14.7%; *k* = 5).

For the fourth domain (‘Measurement of the outcome’), the majority of studies were judged to be at low risk of bias (85.3%; *k* = 29). The remaining studies were considered to be at high risk (14.7%; *k* = 5).

Finally, concerning the fifth domain: ‘Selection of the reported result’, the studies were judged with high risk of bias (26.5%; *k* = 9), some concerns (67.6%; *k* = 23) and low risk of bias (5.9%; *k* = 2).

Among the five domains, the median of low risk rates was 61.8% (14.7 to 94.1%), the median of some concerns rates was 5.9% (0 to 67.6%), and the median of high risk rates was 5.9% (0% to 85.3%).

### Meta-analysis results

*a) Meta-regressions on all studies*

For the meta-analysis, two studies were not included because data were not available to calculate the effect sizes (Blaes et al., 2016; Carlson et al., 2016). In the 32 remaining studies, nine (28%) contained an active control group and 23 (72%) a passive control group. As mentioned before, we decided to use the active group for the three studies with both, active and passive control groups. For the study with two active control groups, we included ‘Ambulant Activity Feedback therapy’ over ‘Psychoeducation’ in the analysis because the first one was supervised by a physiotherapist and not the second one.

Moreover, for four studies, two measures of fatigue were available. For Kim et al. (2013), we chose the Revised Piper Fatigue scale (PFS) because the Medical Outcomes Study Short Form 12-Item Health Survey (SF-12) only included a single item relating to fatigue. Concerning Lengacher et al. (2012), we decided to keep the fatigue item of the M.D. Anderson Symptom Inventory which seemed more informative than the fatigue cluster item which combined different items (fatigue, drowsiness and disturbed sleep). Finally, for two studies (Johns et al., 2015; Lengacher et al., 2016), the fatigue severity score was used and not the fatigue interference score of the Fatigue Symptom Inventory (see method section).

First, we conducted a meta-analysis without any moderator specified in the model (Table 3). The model was already significant (t(31) = 4.04; p = .0003) with the MeBI groups being less fatigued after the intervention than the control groups (*g* = 0.62, I^2^ = 91.51%).

**Table 3:** Meta-analyses with or without moderators with Knapp-Hartung correction.


MODERATORS	*g*	95% CI	STATISTIC (df)	P-VALUE	TAU^2^	I^2^

**No moderator**	0.62	0.30:0.93	t(31) = 4.04	**<.001**	.59	91.51

**Control group moderator**	/	/	F(1,30) = 7.64	**<.01**	.44	88.83

*Active control group*	0.03	–0.48:0.54	t(30) = 0.10	.92	/	/

*Passive control group*	0.83	0.22:1.43	t(30) = 2.76	**<.01**	/	/

**MeBI moderator**	/	/	F(3,28) = 1.03	.33	.58	91.27

*Mindfulness*	0.79	0.35:1.23	t(28) = 3.70	**<.001**	/	/

*Compassion or Mindfulness and Compassion*	–0.26	–1.06:0.54	t(28) = –0.66	.51	/	/

*Other*	0.10	–1.04:1.23	t(28) = 0.17	.86	/	/

*Remotely*	–0.68	–1.52:0.16	t(28) = –1.65	.11	/	/

**Pathology moderator**	/	/	F(5,26) = 0.51	.77	.66	92.58

*Cancer*	0.74	0.33:1.14	t(26) = 3.73	**<.001**	/	/

*Multiple Sclerosis*	–0.48	–1.32:0.37	t(26) = –1.16	.26	/	/

*Fibromyalgia*	0.26	–1.21:1.73	t(26) = 0.36	.72	/	/

*Acute brain injury*	0.03	–1.97:2.03	t(26) = 0.03	.98	/	/

*Myalgic Encephalomyelitis*	–0.23	–2.08:1.63	t(26) = –0.25	.80	/	/

*Kidney Disease*	–0.95	–2.84:0.93	t(26) = –1.04	.31	/	/


*Note*: Meta-analyses on 32 studies. *g*: Hedges’ Standardized Mean Difference (effect size, positive value = less fatigue), CI: Confidence Interval, MeBI: Meditation-Based Interventions.

Then, we used three moderators for meta-regressions: type of control group (active, passive), type of MeBI (mindfulness, compassion, remotely, other), and pathological condition (cancer, multiple sclerosis, myalgic encephalomyelitis, fibromyalgia, brain injury and kidney disease) to better understand the effect of the MeBI intervention on fatigue. These moderators were examined in separate models (Table 3). When the control group was included as moderator, the model was significant (F(1,30) = 7.64; p < .01), with a significant larger effect of MeBI in studies with a passive control group (*g* = 0.83; CI = [0.22, 1.43]) compared to studies with an active control group (*g* = 0.03; CI = [–0.48, 0.54]). However, this was not the case for pathology or MeBI moderators (ps > .05).^2^

The forest plot of the different studies and their standardized mean differences are presented in Figure 3. Visual inspection shows that zero is comprised in the confidence interval for negative standardized mean difference values, indicating that the effect of the control group was never significantly superior to that of the MeBI.

*b) Exploratory analyses*

We performed subgroup analyses to assess specifically the effect of each moderator (type of control group, MeBI and pathological condition; Table 4).

**Table 4:** Subgroups analyses with Knapp-Hartung correction to assess the effect of each moderator (type of control group, MeBI and pathology) separately.


MODERATORS	GROUPS	K	*g*	95% CI	STATISTIC (df)	P-VALUE	TAU^2^	I^2^

**Type of control groups**	*Passive*	23	0.87	0.47:1.27	t(22) = 4.55	**<.001**	0.68	92.01

	*Active*	9	0.04	–0.15:0.22	t(8) = 0.49	0.64	0.01	23.40

**Types of interventions**	*Mindfulness*	16	0.80	0.28:1.32	t(15) = 3.29	**<.01**	0.80	94.54

	*Compassion or Mindfulness and Compassion*	7	0.52	–0.17:1.21	t(6) = 1.86	.11	0.39	83.36

	*Remotely*	6	0.05	–0.45:0.54	t(5) = 0.24	.82	0.09	59.71

	*Other*	3	0.91	–1.57:3.39	t(2) = 1.58	.25 ^a^	0.78	88.81

**Types of pathologies**	*Cancer*	21	0.75	0.28:1.22	t(20) = 3.34	**<.01**	0.91	94.61

	*MS*	6	0.29	0.09:0.48	t(5) = 3.83	**<.05**	0.00	0.00

	*Fibromyalgia*	2	1.05	–2.44:4.55	t(1) = 3.83	.16 ^a^	0.00	0.00

	*Acute brain injury*	1	0.77	–0.05:1.58	Z = 1.84	.07 ^a,b^	0.00	0.00

	*Myalgic Encephalomyelitis*	1	0.52	0.09:0.93	Z = 2.37	**<.05** ^a,b^	0.00	0.00

	*Kidney Disease*	1	–0.21	–0.75:0.32	Z = –0.79	.43 ^a,b^	0.00	0.00


*Note*: ^a^ P-values are not reliable when df < 4, ^b^ Hartung-Knapp correction could not be applied. [k: Number of studies; *g*: Hedges’ Standardized Mean Difference (effect size, positive value = less fatigue); CI: Confidence Interval; MS: Multiple Sclerosis].

We first measured the impact of MeBI on fatigue in studies that used a passive control group and those using an active control group. For passively controlled studies, MeBI outperformed comparators with a large effect size (*k* = 23; *g* = 0.87; CI = [0.47, 1.27]; *p* < .001; I^2^ = 92.01%). However, for actively controlled studies, the between group difference was not significant (*k* = 9; *g* = 0.04; CI = [–0.15, 0.22]; *p* = .64; I^2^ = 23.40%).

Next, we investigated the impact of different MeBI types on fatigue. There was a significant effect of mindfulness-based interventions with a large effect size (*k* = 16; *g* = 0.80; CI = [0.28, 1.32]; *p* = <.01; I^2^ = 94.54%). However, we did not find an effect for compassion interventions or mindfulness-based and compassion interventions (*k* = 7; *g* = 0.52; CI = [–0.17, 1.21]; *p* = .11; I^2^ = 83.36%), or for interventions delivered remotely (*k* = 6; *g* = 0.05; CI = [–0.45, 0.54]; *p* = .78; I^2^ = 59.71%). Concerning ‘other’ MeBIs (k = 3), there are not enough studies (Table 4, *df* < 4) to consider the results as reliable.

For the impact of MeBI on fatigue in different pathological conditions, a positive effect of MeBI on fatigue with a large effect size was observed for cancer pathology (*k* = 21; *g* = 0.75; CI = [0.28, 1.22]; *p* = <.01; I^2^ = 94.61%), while a small effect size was observed for multiple sclerosis (*k* = 6; *g* = 0.29; CI = [0.09, 0.48]; *p* = .001).

Concerning fibromyalgia (k = 2), myalgic encephalomyelitis (k = 1), kidney diseases (k = 1), acute brain injury (which was also the only study using a mental fatigue score), there are not enough studies (Table 4, *df* < 4) to consider the results as reliable.

*c) Sensitivity analyses*

The publication bias analysis cannot be assessed as we did not find unpublished studies on the topic. Due to a high heterogeneity between studies, supplementary analyses were performed without outliers (studies with the largest positive effect of meditation, k = 6). The meta-analysis without moderator remains significant. For the meta-regression, we obtain similar results with the 26 remaining studies: a significant effect of the type of control group but not of the type of MeBI or the type of pathology (See *Supplemental Table 4*). Concerning the subgroup analyses, we still observe a higher reduction of fatigue for MeBI compared to passive control groups, a higher reduction in the Mindfulness group and also for people that had cancer or multiple sclerosis (See *Supplemental Table 5*). However, there is now also a significant effect of MeBI by comparison with active control groups.

## Discussion

We synthesized 34 randomized studies that assessed the impact of MeBIs on fatigue in adults with pathological conditions. Concerning the studies’ characteristics, the most frequent population type was cancer. Most of the studies tested the impact of a mindfulness-based intervention. The interventions lasted between six and twelve weeks. The most frequently used scale was the Fatigue Symptom Inventory. Regarding the participants, they were mainly middle-aged women, likely reflecting the age at which people typically develop the pathological conditions under study. Among the 16 studies that used a follow-up and showed a positive immediate effect, nine studies showed that the effect of the intervention was maintained across time, among which seven reported a larger decrease of fatigue in MeBIs compared to control groups.

The meta-analysis without moderator showed an effect of MeBIs on fatigue compared to control groups. More precisely, the meta-regressions with moderators showed that MeBIs outperform passive comparators, with a large effect size. This suggests that following a MeBI is better for reducing fatigue than being on a waiting list or receiving usual care. MeBIs are known to affect both physical and mental health, notably by reducing inflammatory processes (Shakhar et al., 2007), relieving anxiety and depression states, and decreasing pain symptoms (Hofmann et al., 2010; Marchant et al., 2021; Morone et al., 2008; O’Connor et al., 2014). Inflammatory processes constitute the primary cause of fatigue in pathological disease while pain, anxiety and depression states would represent secondary causes (Penner & Paul, 2017). Therefore, meditation-based intervention could help reducing fatigue by alleviating directly this symptom but also by reducing other pathology-related symptoms that can trigger fatigue.

However, we did not observe a significant effect of MeBIs when comparing them to actively controlled studies in our main analysis. The absence of significant difference between MeBIs and active control groups could be explained by the importance of social interactions and (un)formal support provided by all kind of interventions and their benefits on quality of life (Kroenke et al., 2013). An alternative explanation is that non-pharmacological interventions, and more particularly CBT, have either direct benefits on fatigue or an indirect one through emotional variables (Van Kessel et al., 2008). Indeed, the majority of active control groups included in this review contained psychoeducation. In the meta-analysis, the groups were: education or psychoeducation (k = 5), support (k = 2), music (k = 1), physical activity (k = 1), muscle relaxation (k = 1). In the systematic review, there was also another support group (k = 1).

Regarding publication bias, only five studies on meditation and fatigue were reported on OpenGrey, three on healthy people, one was qualitative study, and one was preliminary results presented in a doctoral dissertation and published later (Hoffman et al., 2012). This could represent a publication bias although we cannot also exclude the possibility that this topic is under-represented in the literature.

Regarding the intervention moderator, the mindfulness interventions appear to be interesting to use for the alleviation of fatigue symptoms. Due to the higher number of studies on the topic, we also looked at subgroups as exploratory analyses (Table 4), but the effect remains. Concerning remotely delivered MeBIs, the absence of significant effect could be explained by a lack of social interactions in some studies, as well as the difficulty to really understand the techniques. For example, three studies (Bruggeman-Everts et al., 2017; Kubo et al., 2019; Messer et al., 2019) used audio files and not movie files (e.g., learning of respiration techniques could be easier by observing someone). The remaining studies included the possibility to interact with the teacher in addition to video- (Bogosian et al., 2015; Cavalera et al., 2019) or tele-conference (Gross et al., 2017). However, the small number of studies makes it impossible to assess the effect of discussion with the teacher.

Regarding the moderator of pathological condition, MeBI seems to have a strong impact on fatigue related to cancer. Again, the number of studies being higher for this population, we decided to look at subgroup analyses (Table 4) and we found that the effect remains for people with cancer but there was also an effect for people with multiple sclerosis. Whilst MeBI also reduced fatigue in myalgic encephalomyelitis, the number of relevant studies in each condition is sparse. Kim et al. (2013) reported that fatigue decreased in the meditation group but increased in the control group when patients are treated with radiation therapy. However, it seems that MeBIs did not just stabilize the level of fatigue induced by radiation but also reduced fatigue compared to baseline. This is particularly interesting as there is no clear evidence of a positive effect of pharmacological treatment on fatigue (see for example Payne, Wiffen, & Martin, 2012 cited by Penner & Paul, 2017).

From a theoretical viewpoint, mental and physical fatigue are considered as distinct entities (Penner & Paul, 2017), with the former corresponding to a sensation of mental exhaustion. Only one trial specifically evaluated the efficacy of MeBIs on mental fatigue and reported a large effect size (Johansson et al., 2012). All other studies in this review included measures of general fatigue (combining mental and physical fatigue reports). The effect of MeBIs on mental fatigue is thus an under-investigated topic, particularly concerning pathological conditions associated with an important fatigue complaint. However, in healthy individuals, MeBI seems also positively influence mental fatigue (see for example Coimbra et al., 2021; Kudesia, Pandey, & Reina, 2022).

To sum up, our systematic review and meta-analysis shows a positive effect of MeBIs on fatigue in pathological conditions. Exploratory analyses showed that mindfulness MeBI seems the most helpful, particularly in cancer and multiple sclerosis patients. This confirms the results of three previous recent systematic reviews on cancer (Castanhel & Liberali, 2018; Haller et al., 2017; Johns et al., 2021). However, further studies are necessary to confirm these first evidence. For example, in fibromyalgia, myalgic encephalomyelitis, kidney diseases, as well as acute brain injury, only one or two studies exist. Moreover, these studies were interested in global fatigue, with only one study discussing the effect on mental fatigue. This is particularly crucial now due to the COVID-19 pandemic. Indeed, as mentioned in the introduction, fatigue complaints are very frequently expressed in long-COVID patients (Ceban et al., 2022; Stavem et al., 2021). Moreover, it would also be interesting to conduct mediation analyses to see if there is a direct impact of meditation on fatigue or/and if meditation impacts on other factors linked to fatigue (anxiety, stress, depression, and pain).

A still-open question is how the intensity of the fatigue could affect MeBI efficacy. Some studies therefore used a specific level of fatigue in their inclusion criteria. For example, Johns et al. (2015) showed a significant effect on a population with a high fatigue level at screening. However, other studies that did not use fatigue intensity as inclusion criteria do not report a positive effect of the MeBI (e.g., Speca et al., 2000). In this review, six studies included only people with a high level of fatigue (Bruggeman-Everts et al., 2017; Johansson et al., 2012; Johns et al., 2015; Johns et al., 2016; Rimes & Wingrove, 2013; Van Der Lee & Garssen, 2012). Among these six studies, four included a passive control group and showed a positive effect of MeBI on fatigue while this is not observed by comparison to active control (*k =* 2) on fatigue to an active control group. Further studies are necessary to determine if the effect of MeBI is driven by fatigue level.

Finally, our review gathered articles employing a subjective assessment of fatigue. A recent meta-analysis showed a beneficial effect of MeBIs on objective measures of cognition (Whitfield et al., 2021). Future studies are needed to establish the impact of MeBIs on fatigue as measured by behavioral outcomes (e.g., decrease of performance on long, difficult or highly demanding cognitive tasks or physically demanding tasks (see for example Borragan et al., 2016, 2017; Johnson et al., 1997; Paul et al., 1998; Wang et al., 2016). Moreover, physiological parameters such as pupil dilatation also seem interesting to obtain objective (i.e., not self-reported) measures of fatigue (Guillemin et al., 2022; Hopstaken et al., 2015). However, these behavioral and physiological measures of fatigue can be influenced by boredom or lack of motivation and there is an interest to control for these aspects.

## Strengths

The major strength of this systematic review and meta-analysis is that we included considerably more studies with different pathological conditions than in previous publications. Eight databases were searched for unpublished studies and we finally included 34 studies yielding the most comprehensive review of MeBIs on fatigue in pathological conditions. Meta-regressions were done on 32 studies, three moderators were used and exploratory subgroups analyses were also run. Therefore, the current results are likely to be more reliable, especially concerning the meta-analysis. We also included more pathological conditions across which effects could be contrasted, and compared several types of MeBI as well.

## Limitations

First, there is a lack of homogeneity among fatigue-related data. Indeed, 16 different scales were administered among the included studies. The three components of pathological fatigue (severity, persistence and impact on daily functioning (Christodoulou, 2005)) are included in the Fatigue Severity Scale, while other scales just assessed one or two components. Second, even if we made a qualitative distinction between the different pathological conditions, comparing them quantitatively would be difficult due to the small number of studies conducted in each population (i.e., one study on fibromyalgia, one on acute brain injury, one on kidney disease). Third, some characteristics of participants can differ between studies. Indeed, age, medication, and daily activities can vary among different study groups.

## Implications

As mentioned previously, fatigue is highly debilitating in various pathologies. Because of its severity and frequency, it is crucial to consider and try to mitigate this state. Fatigue is typically not effectively treated using pharmacological interventions. A potential useful non-pharmacological approach could thus be MeBI.

Alleviating the symptoms of fatigue will positively affect everyday life among people with pathological conditions. Indeed, different studies have shown that fatigue can interfere with work, activities and social life (Nowe et al., 2019; Penner & Paul, 2017). Therefore, according to the results of this systematic review and meta-analysis on more than 30 studies and seven pathologies, we consider that MeBIs could contribute to alleviating fatigue, thereby improving quality of life, well-being at work and in personal life, and health issues in patients with disease leading to disabling fatigue.

## Conclusion

This study aimed to identify the impact of MeBIs on fatigue in pathological conditions. We showed that people who followed a MeBI experienced a greater reduction in fatigue symptoms compared to passive control groups. However, the change in levels of fatigue did not differ between meditation and active control groups. It seems particularly relevant to use MeBIs to cope with fatigue in cancer and multiple sclerosis. The studies about other pathological conditions are scarce, making it difficult to reach conclusions. Further studies are needed to more comprehensively evaluate the impact of various types of MeBI on various pathologies associated with debilitating fatigue.

## Additional File

The additional file for this article can be found as follows:

10.5334/pb.1182.s1 Supplemental Material. Information about study design (prevalence of scales and groups used in all the studies), follow-up data as well analysis without outliers can be retrieved in Supplemental Material.

## Footnotes

Loving-kindness and compassion states will be referred here as “compassion interventions” for ease of reading.

We decided to also run the analyses by excluding the fourth category of MeBis (“Others”) due to their weak empirical support. No changes in our models were observed and therefore these interventions were kept in our general model.
