# Supplementary material for: The Effect of Meditation-Based Interventions on Patients with Fatigue Symptoms: A Systematic Review and Meta-Analysis
Source: Psychol Belg. 2023 Jun 23;63(1):64–81. doi: 10.5334/pb.1182 (PMC10289041; doi:10.5334/pb.1182)
Supplement: Supplemental Material. — Information about study design (prevalence of scales and groups used in all the studies), follow-up data as well analysis without outliers can be retrieved in Supplemental Material. [file pb-63-1-1182-s1.pdf]

Supplemental Material - "The effect of meditation-based interventions on patients with fatigue symptoms: a systematic review and meta-analysis."

Table of Contents

List of tables

- **Supplemental Table 1.** Fatigue scales used in the 34 studies included
- **Supplemental Table 2.** Types of meditation-based intervention (MeBI) categories
- **Supplemental Table 3.** Follow-up data for the 16 studies showing an immediate effect of a meditation-based intervention
- **Supplemental Table 4.** Sensitivity analyses with Knapp-Hartung correction: meta-analyses with and without moderators (without outliers)
- **Supplemental Table 5.** Sensitivity analysis with Knapp-Hartung correction (subgroups analyses without outliers): specific effect of each moderator (type of control group, MeBI and pathology)

Supplemental Table 1. Fatigue scales used in the 34 studies included

Number of studies (N=34)	Fatigue scales (Total=17)	Types of fatigue scores measured by the scale
6	**Fatigue Symptom Inventory (FSI)**	*Perceived severity, frequency, and interference with daily functioning (subscales)*
4	**The Profile of Mood States (POMS)**	*Fatigue-inertia (1 item)*
4	**Brief Fatigue Inventory (BFI)**	*Global fatigue (total score)*
2	**Checklist Individual Strength (CIS)**	*Subjective fatigue, Concentration, Motivation, Physical Activity (subscales)*
3	**Modified Fatigue Impact Scale (MFIS)**	*Mental, physical, and psychosocial fatigue (subscales)*
2	**Quality of Life Questionnaire Core 30 Items (QLQ-C30)**	*Fatigue (1 item)*
2	**Chalder Fatigue Scale**	*Physical and mental fatigue (subscales)*
2	**Fatigue Severity Scale (FSS)**	*Global fatigue (total score)*
2	**Patient-Reported Outcomes Measurement Information System (PROMIS)**	*Fatigue (1 item)*
1	**FACIT Fatigue scale**	*Global fatigue (total score)*

1	**Revised Piper Fatigue scale (PFS)**	*Behavioral/severity, affective meaning, sensory, and cognitive/mood (subscales)*
1	**Medical Outcomes Study Short Form 12-Item Health Survey (SF-12)**	*Vitality-fatigue (1 item)*
1	**M.D. Anderson Symptom Inventory (MDASI)**	*Fatigue (1 item)*
1	**Multidimensional Fatigue Scale Inventory – Short Form (MFSI-SF)**	*Mental, physical, and psychosocial fatigue (subscales)*
1	**Functional Assessment in Cancer Therapy-Fatigue (FACT-F) European**	*Global fatigue (total score)*
1	**Mental Fatigue Scale (MFS)**	*Mental Fatigue (total score)*

Note. All the studies reported a total score including physical and mental fatigue except one that used Mental Fatigue Scale.

Supplemental Table 2. Types of meditation-based intervention (MeBI) categories

**MeBI categories**	**Total**	**Used in the meta-analysis**
Mindfulness-Based Intervention	18	**16**
Compassion interventions or Mindfulness and Compassion interventions	7	7
Delivered remotely	6	**6**
Others (*Tibetan Sound Meditation program, Brain Wave Vibration meditation, Mindfulness-Based Art Therapy)*	3	3
**General total**	**34**	**32**

Supplemental Table 3. Follow-up data for the 16 studies showing an immediate effect of a meditation-based intervention

Studies (authors, date)	Number of weeks between intervention and follow-up	Effect of the intervention at follow-up
(Blaes et al., 2016) [b]	9	No
(Bower et al., 2015b)	13	
(Carlson et al., 2016)	56	
(Cash et al., 2015) [a,b]	9	
(Dodds et al., 2015)	4	
(Gok Metin et al , 2019)	2	
(Hoffman et al., 2012)	6 to 8	
(Liu et al., 2019)	14	
(Witek Janusek et al., 2019)	52	
(Bogosian et al., 2015)	20	Yes
(Grossman et al., 2010)	26	
(Johns et al., 2015)	4	
(Lengacher et al., 2016)	6	

(Meiklejon, 2008)	13
(Rimes & Wingrove, 2013)	9
(Van Der Lee & Garssen, 2012)	26

Note. [a] all groups showed an immediate positive effect of the intervention, with no between-groups difference, [b] all groups showed a positive effect of the intervention at follow-up, with no between-groups difference

Supplemental Table 4. Sensitivity analyses with Knapp-Hartung correction: meta-analyses with and without moderators (without outliers)

Moderators	*g*	95% CI	Statistic (df)	p-value	Tau^2	I^2
**No moderator**	0.38	0.25:0.50	t(25)=6.12	**<.0001**	.02	27.85
**Control group moderator**	/	/	F(1,24)=7.96	**<.01**	.00	0.01
**MeBI moderator**	/	/	F(3,22)=0.48	0.70	.03	38.73
**Pathology moderator**	/	/	F(4,21)=1.23	.33	.02	27.52

Note. Meta-analyses on 26 studies. *g*: Hedges' Standardized Mean Difference (effect size, positive value = less fatigue), CI: Confidence Interval, MeBI: Meditation-Based Interventions.

Supplemental Table 5. Sensitivity analysis with Knapp-Hartung correction (subgroups analyses without outliers): specific effect of each moderator (type of control group, MeBI and pathology)

**Moderators**	**Groups**	**k**	***g***	**95% CI**	**df**	**p-value**	**Tau²**	**I² (%)**
**Control groups**	*Passive*	19	0.48	0.33:0.63	t(18)=6.65	**<.0001**	0.02	29.47
	Active	7	0.14	0.02:0.27	t(6)=2.75	**<.05**	0.00	0.00
**Types of interventions**	*Mindfulness*	14	0.44	0.26:0.62	t(13)=5.15	**<.001**	0.04	44.67
	Compassion or Mindfulness and Compassion	6	0.30	-0.07:0.67	t(5)=2.08	.09	0.05	40.40
	Remotely	4	0.22	-0.35:0.79	t(3)=1.23	.31 [a]	0.00	0.00
	Other	2	0.46	-1.27:2.19	t(1)=3.36	.18 [a]	0.00	0.00
**Types of pathologies**	*Cancer*	16	0.38	0.19:0.56	t(15)=4.37	**<.001**	0.04	47.97
	MS	6	0.29	0.09:0.48	t(5)=3.83	**<.05**	0.00	0.00
	Fibromyalgia	2	1.05	-2.44:4.55	t(1)=3.83	.16 [a]	0.00	0.00

Acute brain injury	1	0.77	-0.05:1.58	Z=1.84	.07 [a,b]	0.00	0.00
Myalgic Encephalomyelitis	1	0.52	0.09:0.93	Z=2.37	**<.05** [a,b]	0.00	0.00

Note. [a] P-values are not reliable when df < 4, [b] Hartung-Knapp correction could not be applied. [K: Number of studies; *g*: Hedges' Standardized Mean Difference (effect size, positive value = less fatigue); CI: Confidence Interval; MS: Multiple Sclerosis].
